# Respiratory Syncytial Virus Persistence in Macrophages Alters the Profile of Cellular Gene Expression

**DOI:** 10.3390/v4123270

**Published:** 2012-11-22

**Authors:** Evelyn Rivera-Toledo, Beatríz Gómez

**Affiliations:** Department of Microbiology and Parasitology, Faculty of Medicine, Universidad Nacional Autónoma de México, Circuito exterior s/n, Ciudad Universitaria, México D.F., C.P. 04510, Mexico; Email: begomez@servidor.unam.mx

**Keywords:** Respiratory syncytial virus, viral persistence, macrophages, P388D1, altered gene expression

## Abstract

Viruses can persistently infect differentiated cells through regulation of expression of both their own genes and those of the host cell, thereby evading detection by the host’s immune system and achieving residence in a non-lytic state. Models *in vitro* with cell lines are useful tools in understanding the mechanisms associated with the establishment of viral persistence. In particular, a model to study respiratory syncytial virus (RSV) persistence in a murine macrophage-like cell line has been established. Compared to non-infected macrophages, macrophages persistently infected with RSV show altered expression both of genes coding for cytokines and trans-membrane proteins associated with antigen uptake and of genes related to cell survival. The biological changes associated with altered gene expression in macrophages as a consequence of persistent RSV infection are summarized.

## 1. The Virus: Characteristics, Pathogenesis, and Epidemiology

Respiratory syncytial virus (RSV; family Paramyxoviridae, genus Pneumovirus) is a highly infectious agent—more so than other respiratory viruses—and worldwide is the principal cause of serious lower-respiratory tract illness in infants and young children [1]. Structurally, RSV is an enveloped and pleomorphic virus, with a single-stranded, negative-sense RNA genome encoding 11 proteins [1,2]. Epidemiological studies of RSV indicate that this pathogen is frequently isolated from children with bronchiolitis [3,4] and is the most frequent cause of hospitalization of infants in industrialized countries [5]. Risk factors, such as premature birth, congenital heart disease, and immune deficiencies, predispose children <6 months of age to severe respiratory disease, thus increasing the frequency of RSV-related hospitalizations by as much as 56% [6,7,8]. Most infants experience RSV infection during the first year of life and there exists an association between early severe RSV infection and recurrent wheezing or asthma in later childhood [9,10,11]. RSV is also an important cause of morbidity and mortality in the elderly and in immunocompromised patients [12,13]. In the elderly, RSV is the second leading cause of viral death, with an annual incidence up to 5% [14]. The World Health Organization (WHO) reports 64 million cases and 160,000 deaths each year due to RSV—more than that caused by any other respiratory virus [15]. Seasonal RSV outbreaks occur each year throughout the world during the winter months: in the northern hemisphere, the annual epidemics normally start in November, peak in January and February and end in May; in the southern hemisphere, the epidemic season runs from May through September [16,17].

Prospective studies of cohorts of patients with chronic obstructive pulmonary disease (COPD) have revealed, through reverse-transcription polymerase chain reaction (RT-PCR), that RSV is the virus most frequently detected in nasopharyngeal aspirates during stable COPD and exacerbated episodes [18,19]. The effects of the sequelae of severe RSV disease may be explained, in part, by viral persistence, with the RSV infection causing an alteration of the airway structure and/or inducing an aberrant immune response [9,10,19]. Continuous stimulation of the immune system by persistent viral infections may cause chronic inﬂammation or alter the expression of immunoregulatory molecules [20,21,22]; such outcomes may explain the clinical manifestations that persist long after acute viral infection. Infected epithelial cells and macrophages secrete cytokines, chemokines, and other factors that attract lymphocytes and other cells to the site of infection, thus resulting in airway inﬂammation [23,24].

## 2. RSV Persistence

Although RSV persistence in humans has not been demonstrated, some observations indicate that this may be the case: 1) the presence of RSV antigen in bone biopsies and in osteoclasts cultured from patients with Paget disease was detected by using immunohistological assays [25]; 2) RSV was isolated repeatedly from the nasopharynx of apparently healthy children [26]; 3) RSV nucleoprotein mRNA was detected in archival postmortem lung tissue from infants, who had died during the summer, without apparent clinical disease having been reported [27]; and 4) RSV genome has been detected in human naïve primary bone marrow stromal cells from adults (6/8) and children (3/3) [28].

Persistent RSV infection has been established *in vivo* in mouse and guinea pig models [29,30,31]. In studies using BALB/c mice, persistent RSV infection has been followed through kinetic studies, revealing that infectious virus can be isolated from bronchioalveolar fluid or lymph nodes only during the first 14 days post-infection, whereas in lung homogenates, viral genomic RNA and mRNA can still be detected after 100 days, even though signs of acute infection have disappeared [30]. In guinea pigs, after resolution of acute bronchiolitis and at 60 days post-infection, viral genomic RNA and RSV proteins, along with polymorphonuclear cell infiltrates, can be detected in lungs by RT-PCR and immunohistochemistry [29]. Although, in these models *in vivo*, the cell type that RSV is able to persistently infect has not been determined, studies *in vitro* indicate that RSV can establish persistent infection in epithelial cells, macrophages and dendritic cells [32,33,34,35,36].

The predominant cell type recovered from bronchioalveolar lavages from children with acute severe lower-respiratory tract symptoms is the alveolar macrophage; these macrophages express RSV antigens along with pro-inflammatory cytokines [37]. Also, experiments with calves acutely infected with bovine respiratory syncytial virus (BRSV), a virus closely related to RSV, indicate that upper and lower airway epithelial cells and alveolar macrophages are target cells for the virus, as they became productively infected [38]. In addition, experiments with isolated human alveolar macrophages have shown that this cell type can support prolonged RSV replication (up to 25 days post-infection) without an apparent effect on cell viability, suggesting that macrophages may be an important reservoir for RSV *in vivo* [39].

Succeeding in a persistent infection depends on the ability of the virus to regulate not only its own genes but also the host genes in order to avoid killing the host cell. This is achieved by an alternative viral strategy of replication and the ability to evade the immunologic surveillance system of the host. In this way, the continuous replication of a virus in a differentiated cell can alter the normal functions of said cell without destroying it; this in turn disturbs the homeostasis of the host, thus producing disease [40].

Given that macrophages are important target cells for RSV and that, once infected, they can support a persistent viral infection, this brief review is focused on alterations in the biological functions of a murine macrophage-like cell line persistently infected with RSV.

## 3. Establishment and Characteristics of a Persistently RSV-Infected Macrophage-Like Culture

A model to study the RSV persistence in macrophages was established by using the murine macrophage-like cell line, P388D1, which was derived from serial passages in mice of an original methylcholanthrene-induced lymphoid neoplasm in a DBA/2 mouse [41]. When this cell line was infected at a multiplicity of infection (m.o.i.) of 1.0 with the prototype RSV Long strain (wild-type RSV), both a low frequency of syncytia and a high percentage of cell death during the first 48 h post infection were observed. Nevertheless, after 72 h, the number of macrophages started to increase and the surviving cells were propagated. In the first few passages, 40%–60% of the cells presented viral antigen on their cell membrane; after cloning the cells by limited dilution and reinfecting the clones at an m.o.i. of 1.0, subsequent passages were stabilized, with a constant viral expression in 90%–95% of the cells being achieved [33]. Currently, after more than 85 passages, this line of macrophages persistently infected with RSV (MφP) continues to express the viral genome: mRNA of the N viral gene is detected by RT-PCR and viral proteins are expressed on the cell membrane, as demonstrated by immunofluorescence [42].

One of the effects of persistent virus infection in immortalized cells is alteration of the viral genome, thus producing viral variants adapted for a prolonged period of replication without killing the host cell [40]. Similarly, the RSV in MφP shows genotypic changes, at least in the viral membrane fusion protein (F), compared to the wild-type RSV [43]. The genotypic change in persistent RSV was associated with a decreased fusogenic activity and was manifested by reduced size and frequency of syncytia, as well as with low extracellular viral titer in Vero cells, an RSV-permissive cell line [43]. When the deduced amino acid sequences of the F protein from the persistent and wild-type RSV were compared, changes in nine amino acids were observed, three of which are adjacent to the cleavage domain and the fusion peptide. The particular changes in the region of the cleavage domain suggest that the processing of the F0 precursor by cellular proteases may not be efficient, thus reducing its membrane fusion capacity. This hypothesis is supported by experiments in which the number of syncytia was augmented approximately five-fold when Vero cells infected with persistent RSV were cultured either in the presence of trypsin or in a low pH environment—conditions that have been shown to improve activation of viral fusogenic proteins [44,45,46]. However, it seems that the efficiency of F0 processing from persistent RSV is cell-line dependent, because when lung carcinoma cells H358 were used as target cells for the same persistent virus, neither the enzymatic nor acidic treatment improved the fusogenic activity; in fact, the fusogenic activity was similar to that obtained without treatment, indicating that the intracellular protease activation of the persistent RSV F protein is less efficient in Vero cells than in H358 cells [43].

## 4. Persistent RSV Infection Alters Macrophage Gene Expression and Biological Activities

Macrophages, important cells of the innate immune system, act as a first-line of defense against invading pathogens and help to initiate T-cell responses by processing and presenting antigens. The non-specific defense function of macrophages depends mainly on their ability to take up particulate material by phagocytosis [47]. Phagocytosis can be mediated either directly by receptors on the macrophages recognizing foreign structures of particles or indirectly by receptors that recognize self-ligands (e.g., when a foreign particle is opsonized by complement or by antibodies) [48,49].

Specific phagocytosis mediated by Fcγ receptors (FcγR) of IgG-opsonized sheep red blood cells is three- to six-fold enhanced in MφP, compared to mock infected macrophages (MφN); this relevant change is likely a consequence of an increased level of expression of FcγRII and FcγRIII in the MφP [50]. Arrevillaga *et al.* [42] showed that non-opsonized phagocytosis is also altered in MφP. In that work, MφP show a decreased efficiency in phagocytizing non-typeable Haemophilus influenzae (NTHi), a pathogen associated with exacerbations of COPD, with bacterial adhesion and ingestion being 1.7- and 11-fold less, respectively, than the values obtained with MφN [42]. This diminished uptake of bacteria by MφP is linked to a reduced expression (~50%) of both the ICAM-1 mRNA and ICAM-1 protein on the cell membrane, the latter serving as a ligand to bind bacteria. Although ICAM-1 is not the only ligand for NTHi, the negative transcriptional regulation of this molecule, as a consequence of the persistent RSV infection, could contribute to inefficient bacterial clearance by macrophages.

**Figure 1:**
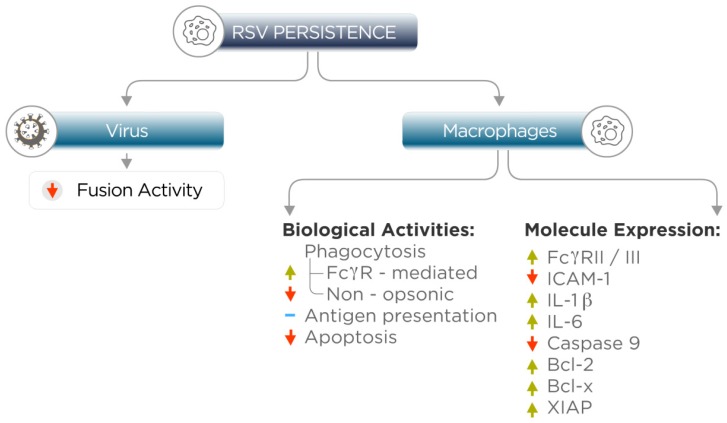
**Changes in respiratory syncytial virus (RSV) and macrophages by persistent infection**. RSV persistence in macrophages leads to genotypic changes, at least in the viral membrane fusion protein F and in the profile of cellular gene expression. Arrows indicate increase or decrease in biological activities or molecule expression.

Dendritic cells, macrophages, and B lymphocytes are “professional” antigen-presenting cells (APCs). Although dendritic cells and their subsets are the most potent stimulators of T lymphocytes, the relevance of particular APCs can be determined according to their abundance in a particular tissue [51]. Alveolar macrophages comprise 95% of the cells of the lung lavage with the remaining portion consisting mostly of leukocytes, thus indicating that macrophages may be important in establishing an early non-specific defense and by functioning as presenting cells to initiate the adaptive immune response in the lung [52]. A study by Guerrero-Plata *et al.* [53], which focused on determining whether MφP preserve their ability to present antigens, showed that persistent infection with RSV increases expression levels of alleles K and D of the MHC class-I molecules to levels similar to those obtained at 24-h post-acute infection. The augmented MHC-I expression in MφP correlates with an efficient processing and presentation of RSV antigens to RSV-specific CD8 T cells, as determined by cytotoxicity assays. Also, MφP maintain the ability to process and present other viral antigens, such as a peptide derived from the influenza virus nucleoprotein (NP147-155). In addition, the profiles of cytokine expression in supernatants of MφP and MφN cultures indicate that the cytokines IL-1β and IL-6 are statistically significantly augmented in the MφP, suggesting that persistent RSV infection keeps macrophages in a permanently activated state [50]. Acute RSV infection of lung epithelial cells and granulocytes induced prolonged survival of infected cells by increasing the expression of anti-apoptotic molecules of the Bcl-2 family [54,55]. MφP, under normal culture conditions, display similar viability as MφN [56]. However, treatment of these macrophage cultures with staurosporine—an inhibitor of protein kinases, which induces cellular apoptosis in the original P388D1 cell line [57]—induces cell death of almost all MφN after 24 h, whereas more than 75% of MφP are refractory [56]. MφP resistance to apoptosis is associated with reduced expression of the protein pro-caspase 9, although its mRNA levels are normal or even higher than in MφN, suggesting that persistent infection regulates caspase 9 expression at a post-transcriptional level. Furthermore, chronic RSV infection of MφP up-regulates mRNA and the protein products of anti-apoptotic genes such as Bcl-2, Bcl-x, and XIAP, indicating that abrogation of the intrinsic pathway of apoptosis is a mechanism crucial for the establishment and maintenance of viral persistence [56]. Figure 1 summarizes changes in virus and MφP as a consequence of persistent infection.

## 5. Relevance of RSV Persistence in Macrophages and Epithelial Cells

Understanding the virus-cell interactions during acute and persistent RSV infections is fundamental for the development of strategies to inhibit viral infection and to eliminate viral reservoirs. Models *in vitro* and *in vivo* have been useful tools in advancing comprehension both of the mechanisms by which RSV establishes persistence and of the pathology associated with chronic infection. Models *in vitro* with macrophages and epithelial cell lines have been particularly useful in determining, at the molecular level, alterations produced in the host cell by long-term RSV infection [32,42,53,56]. To date, in addition to MφP, the only other cell model of persistent infection by RSV, in which changes in cellular gene expression have been studied, are persistently infected HEp-2 epithelial cells. Martínez *et al.* [32] reported that, as determined by microarray analysis, several genes with diverse functional categories were either up- or down-regulated in persistently RSV-infected HEp-2 cells. In particular, it was observed that some of the genes that were up-regulated were those involved in cell survival, such as those encoding for the anti-apoptotic molecules TRAF-1 and BIRC3, and that some of the genes that were down-regulated were pro-apoptotic genes, such as tnf-α, bcl2l11, and caspase 9. In contrast to that in MφP, persistent RSV infection in HEp-2 cells regulates caspase 9 expression at the translational level. The study also showed that, although the chemokines CCL3 and RANTES are up-regulated during acute and persistent RSV infection, the levels of these chemokines in persistently infected HEp-2 cells are up to two-fold greater than those in acutely infected HEp-2 cells. It has also been reported that, in a model of RSV persistence in human epithelial cells A549, the level of the cytokine IL-8, evaluated by ELISA in supernatants, is up to 2.6-fold greater than that in mock-infected cells [34]. Thus, when taken together, the findings 1) that RSV can establish persistent infection in macrophages and epithelial cell *in vitro*, 2) that alterations in gene expression lead to survival of persistently infected cells, and 3) that persistently infected cells produce excessive level of cytokines and chemokines that are associated with chronic inflammation lend strong support to the hypothesis that RSV persistence in patients may be a cause of chronic respiratory diseases. It is still to be determined whether altered expression of membrane molecules related to antigen uptake by macrophages occurs in models *in vivo* and, if so, whether such altered expression is relevant to pathogenesis.

## 6. Conclusion

RSV can productively infect macrophages *in vivo* and *in vitro* and can establish persistent infection in macrophage-like cells *in vitro*. The consequence of persistent RSV infection in macrophages is the altered expression of genes coding for pro-inflammatory cytokines, for trans-membrane proteins related to antigen uptake, and for those proteins related to cell survival. The evidence suggests that macrophages may be one of the cell populations that can serve as viral reservoirs for RSV *in vivo*. Understanding how RSV manipulates host cells during persistent infection may provide important insights into new approaches for rational drug design and vaccines.
